# Anomalous dips in reflection spectra of optical polymers deposited on plasmonic metals

**DOI:** 10.1515/nanoph-2022-0450

**Published:** 2023-01-24

**Authors:** Ayanna Shorter, Md Golam Rabbani Chowdhury, Sangeeta Rout, Mikhail A. Noginov

**Affiliations:** Center for Materials Research, Norfolk State University, Norfolk, VA 23504, USA

**Keywords:** optical polymers, plasmonic metals, reflection spectra, refractive indexes, scattering, surface plasmon polaritons

## Abstract

We have studied reflection spectra of dye-doped and undoped polymers deposited onto Ag and Au substrates and found anomalous dips in the UV spectral range. On top of Ag substrates, the *λ* ∼ 375 nm dips were observed in undoped PMMA, PVP, and PS polymers as well as PMMA doped with Rh590 and HITC laser dyes. In silver-based samples, the spectral positions of the observed reflection dips were close to singularities in the refractive indexes of surface plasmon polaritons (SPPs) propagating at the interface between Ag and polymer. The latter singularities can tentatively explain the *λ* ∼ 375 nm reflection dips, if the scattering of Ag and polymeric films is large enough to launch SPP without any prism or grating. The dips observed in reflection of Rh590:PMMA and HITC:PMMA on top of Au, were more pronounced than those on Ag, broader, shifted to shorter wavelengths, and their spectral positions had large standard deviations. Furthermore, no anomalous dips in gold-based samples were observed in the reflection spectra of undoped PMMA, PVP, and PS polymers, and a modest singularity in the SPP refractive index, predicted theoretically at *λ* ∼ 500 nm, cannot explain the dips in the UV reflection spectra observed experimentally. It appears likely that the origin of the reflection dips on top of Au substrates is different from that on top of Ag substrates.

## 1 Introduction

**Figure 1:**
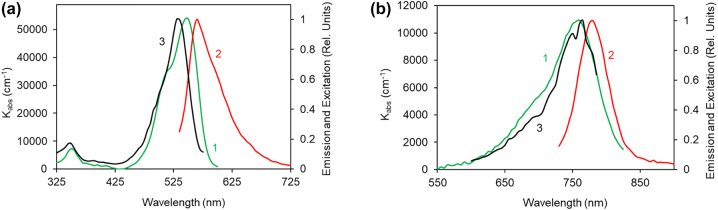
Absorption, emission, and excitation spectra on top of glass. (a) Absorption (trace 1, dye concentration *n* = 128 g/L), emission (trace 2, *n* = 128 g/L), and excitation (trace 3, *n* = 16 g/L) spectra of Rh590:PMMA. (b) Same for HITC:PMMA (trace 1: *n* = 30 g/L; trace 2: *n* = 20 g/L; trace 3: *n* = 30 g/L). (Here and below, the dye concentrations are given for dry polymer, when all solvent evaporated).

Quantum emitters, including dye molecules, play an important role in fundamental studies [1, 2] and applications [3] of nanophotonic and plasmonic materials and devices [4]. Thus, control of emitters’ spectroscopic properties with metal-dielectric environments, including metamaterials [5], metasurfaces [6], Fabry–Perot cavities [7, 8], and Metal-Insulator-Metal (MIM) waveguides [9], has been extensively researched in the literature [10, 11]. Two particularly important and extensively studied laser dyes [12–18], emitting in the visible and near-infrared ranges of the spectrum, are rhodamine 590 chloride (Rh590) and hexamethyl indotricarbocyanin (HITC), respectively. Their absorption, emission, and excitation spectra (Figure 1a and b) are dominated by the S_0_–S_1_ transitions, at *λ* ∼ 530 nm in Rh590 [16, 17] and *λ* ∼ 760 nm in HITC [18]. In Rh590, a weaker S_0_–S_2_ band in the absorption and excitation spectra can be seen at *λ* ∼ 350 nm, Figure 1a. The S_0_–S_2_ transition in the HITC dye is not clearly identified.

**Figure 2:**
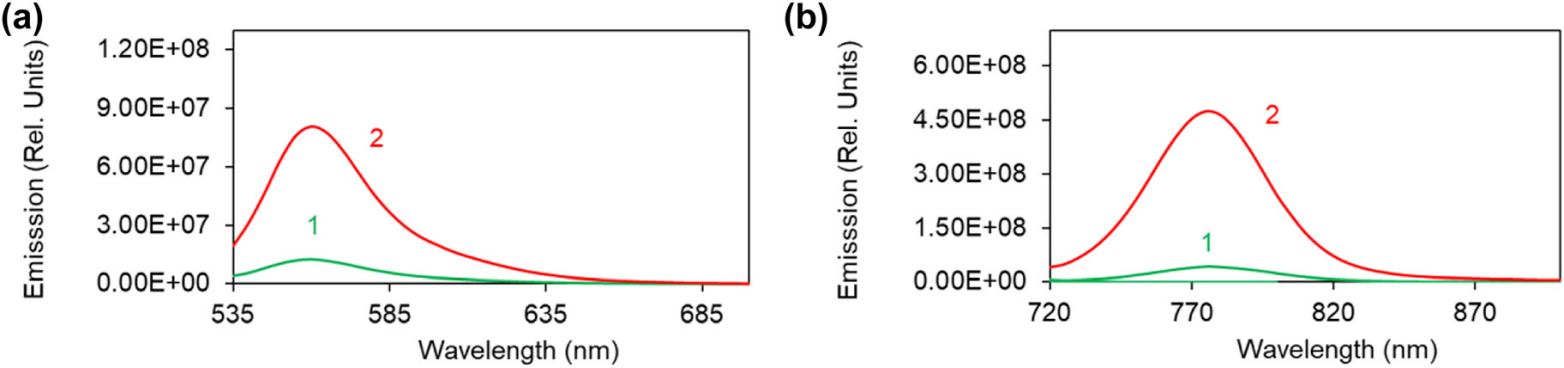
Emission spectra on top of glass, Ag, and Au. (a) Emission spectra of Rh590:PMMA (*n* = 8 g/L) deposited on glass (trace 1) and on Ag (trace 2). The emission spectra are normalized by the absorbed pumping intensity. (b) Same for HITC:PMMA (*n* = 10 g/L) deposited on glass (trace 1) and on Au (trace 2).

When the dye-doped polymers (Rh590:PMMA and HITC:PMMA) are deposited onto Ag or Au substrates, the emission intensity (normalized by the absorbed pumping power) increases up to tenfold, Figure 2a and b. At high dye concentrations, this effect can be explained by inhibition of the concentration quenching (Förster energy transfer to acceptors) in the vicinity of metals [18]. The origin of this phenomenon at low dye concentrations is less clear. This is the subject of the future study to be published elsewhere.

## 2 Anomalous dips in reflection: preliminary observations and motivation

**Figure 3:**
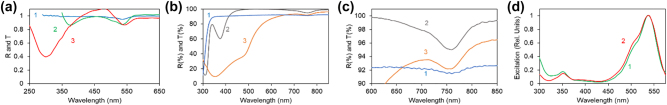
Transmission, reflection, and excitation spectra on top of glass, Ag, and Au. (a) Transmission (1) and reflection (2 and 3) spectra of Rh590:PMMA (*n* = 16 g/L) deposited on glass (1), Ag (2) and Au (3). (b) Same for HITC:PMMA films (*n* = 30 g/L). (c) Zoomed portion of Figure 3b. (d) Excitation spectra of Rh590 emission on top of glass (1) and Ag (2), *n* = 128 g/L.

In our recent study (unpublished) we have found that reflection spectra of thin Rh590:PMMA and HITC:PMMA dye-doped polymeric films deposited on Ag feature strong spectral bands (dips) at *λ* ∼ 375 nm (Figure 3a and b), in addition to the expected S_0_ → S_1_ spectral bands (Figure 3a–c). Qualitatively similar dips, although at shorter wavelengths, have been observed in dye-doped polymers Rh590:PMMA and HITC:PMMA deposited on Au, Figure 3a and b. However, no significant *λ* ∼ 375 nm bands were observed in transmission spectra of the Rh590 and HITC films on top of glass, Figure 3a and b.

The intriguing modification of the spectroscopic properties of dye-doped polymeric films on top of plasmonic metals, described above, which is of high importance to fundamental and applied research fields of nanophotonics, plasmonics, and metamaerials, motivated the present study.

## 3 Sample fabrication

The samples in our experiments were thin films of dye-doped or undoped polymers spin coated onto Ag and Au films or glass substrates. The polymers were poly methyl methacrylate (PMMA), polyvinyl pyrrolidone (PVP), and polystyrene (PS); and the laser dyes were Rh590 and HITC. The metallic films, fabricated using the thermal vapor deposition technique (Nano 36 apparatus from Kurt J Lesker), were ∼130 nm thick. The roughness of the films deposited using a similar apparatus was equal to ∼5 nm [19]. Dyes and polymers were dissolved in dichloromethane (DCM) in a heated sonicated bath for 60 min, after which ∼100 nm thin polymetric films were deposited on the substrates using the Model 6808P spin coater from Specially Coating Systems. The dye concentration in solid state dry polymer ranged from 2 g/L to 512 g/L. The film thickness was measured using the stylus profilometer (DekTak XT, from Bruker). The unpolarized transmission and reflection spectra of polymeric and metallic films were measured (at nearly normal incidence) using the UV–Vis–NIR spectrophotometer, Lambda 900 from PerkinElmer.

## 4 Experimental results

In the particular experiment below, we collected *excitation* spectra of the Rh590:PMMA emission (recorded at *λ* ∼ 600 nm, S_1_ → S_0_) on top of glass and Ag substrates (Figure 3d) and *did not* see any significant bands at *λ* ∼ 375 nm, while we have routinely observed them in the *reflection* spectra measured on top of Ag, Figure 3a. This is the strong evidence that the *λ* ∼ 375 nm band in the reflection spectrum has nothing to do with absorption of Rh590 molecules but has a completely different origin.

**Figure 4:**
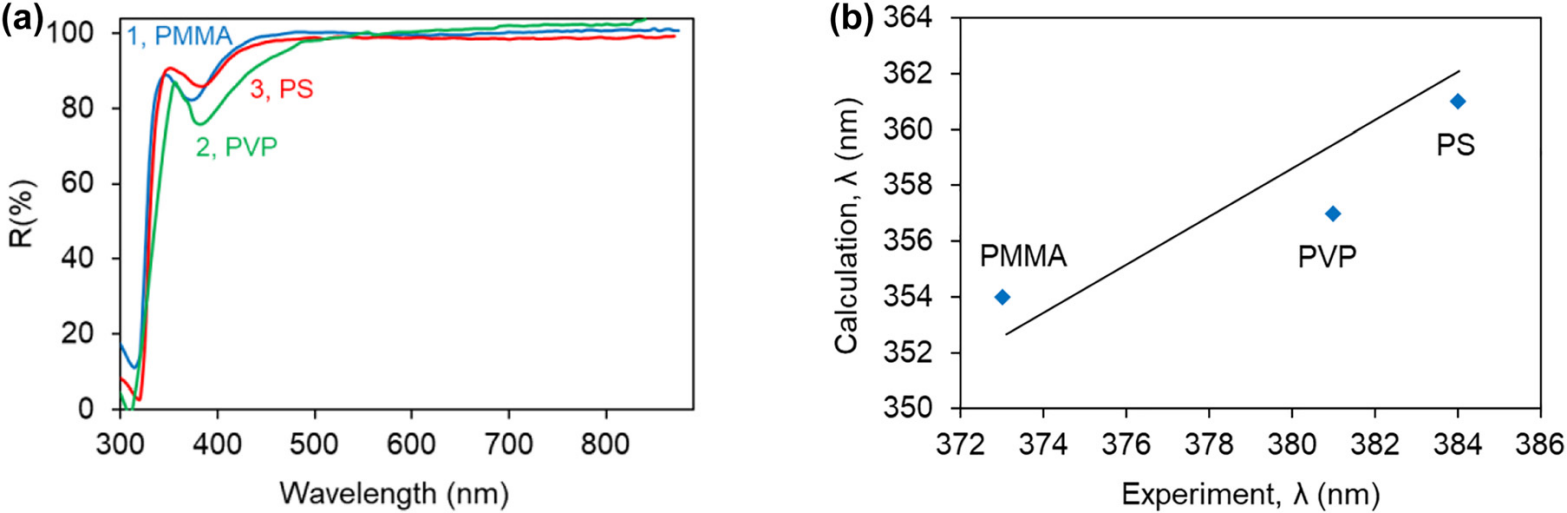
Spectral positions of reflection dips. (a) Reflection spectra of undoped polymers PMMA (1), PVP (2), PS (3) on top of Ag, featuring dips at *λ* ∼ 375 nm. (b) Calculated versus experimental wavelengths of the dips in the reflection spectra.

**Figure 5:**
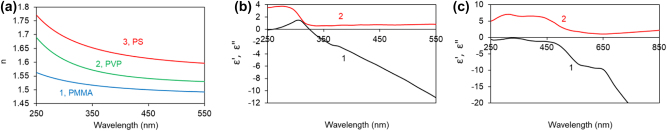
Refractive indexes and dielectric permittivities of polymers, Ag, and Au. (a) Spectra of refractive indexes of PMMA (1), PVP (2), and PS (3). (b and c) Spectra of real (1) and imaginary (2) parts of dielectric permittivity of Ag (b) and Au (c) [20].

The most decisive evidence proving that the *λ* ∼ 375 nm reflection band *was not* due to Rh590 or HITC molecules was obtained when *undoped* PMMA, PVP, and PS polymers were deposited on Ag substrates: all these spectra had *λ* ∼ 375 nm reflection dips (Figure 4a), whose exact spectral positions depended on the refractive index: the smaller the index, the shorter the dip’s wavelength, see Figures 4a and b and 5a.

## 5 Modeling of surface plasmon polaritons

**Figure 6:**
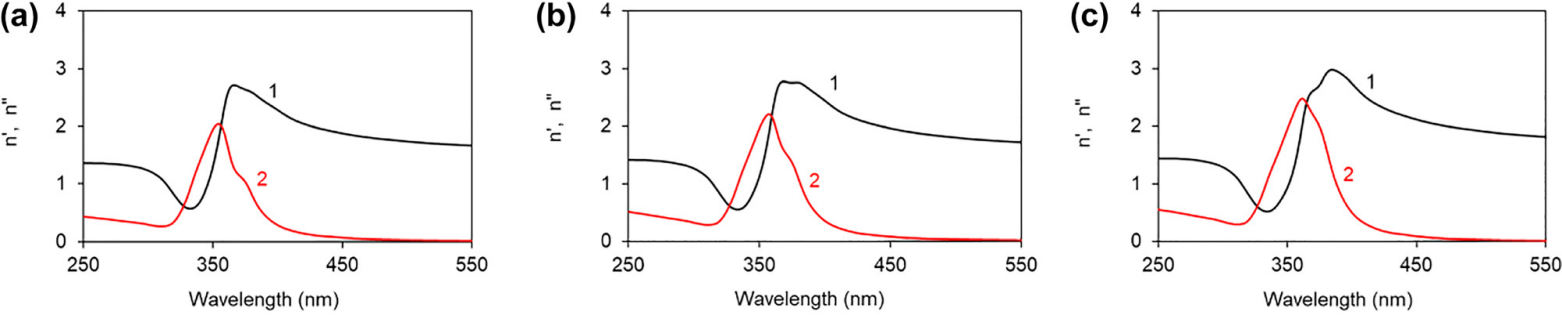
Calculated spectra of real (1) and imaginary (2) parts of refractive indexes of SPPs propagating at the interface between Ag and undoped PMMA (a), PVP (b), and PS (c) [21]. The real and imaginary dielectric permittivities of Ag are adopted from Ref. [20].

The existence of the *λ* ∼ 375 nm dips in the reflection spectra of polymers (with or without dye) deposited on Ag, can be tentatively explained in terms of SPPs propagating at the interface between metal and dielectric (polymer). The effective refractive index of SPPs is given by the formula [22],

(1) $$n_{\text{SPP}}=\sqrt{\frac{\varepsilon_{m}\cdot \varepsilon_{d}}{\varepsilon_{m}+\varepsilon_{d}}}$$

where *ε*_*m*_ and *ε*_*d*_ are the dielectric permittivities of polymers and metals, respectively [22] (Figure 5a–c). We calculated the corresponding SPP dispersion curves and found that real and imaginary parts of *n*_SPP_ have Lorentzian like singularities in vicinity of *λ* ∼ 360 nm, Figure 6a–c, close to the positions of the spectral bands observed in the experimental reflection spectra of polymers on Ag, Figure 4a and b. The order of the above spectral features, *λ*_PMMA_ < *λ*_PVP_ < *λ*_PS_, was the same as the order of the experimental dips in the reflection spectra, Figure 4a, and the order of refractive indexes of the same three polymers in the UV range of the spectrum, *n*_PMMA_ < *n*_PVP_ < *n*_PS_ (Figures 4b and 5a). We, thus, tentatively conclude that the dips in the reflection spectra of the polymers on top of Ag are due to singularities in *n*_SPP_ occurring when the real part of the denominator in Eq. (1) is equal to zero. The modest disagreement between the spectral positions of the experimental (*λ* ∼ 375 nm) and calculated (*λ* ∼ 360 nm) spectral bands, Figure 4b, can be due to the fact that the dielectric permittivities in our experiment were not exactly the same as those used in the calculations [20, 21]. The further mismatch can be caused by the fact that Eq. (1) is valid for semi-infinite metal and dielectric, and the polymer film thickness in our experiment was only 100 nm.

**Figure 7:**
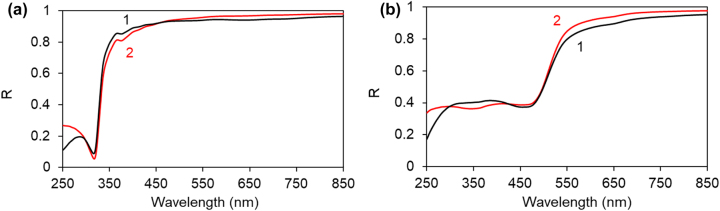
Calculated reflection spectra. (a) The reflection spectrum of Ag substrate with (1) or without (2) 100 nm PMMA on top, calculated using the transfer matrix solver [23]. (b) Same for Au substrate. (*n* of PMMA was assumed to be equal to 1.49).

The key question pertaining to the explanation above is how the SPP was excited without any prism or grating. Not surprisingly, no significant dips in the reflection spectra have been theoretically predicted using the transfer matrix solver assuming perfectly smooth metallic and dielectric layers [23], Figure 7a. The small singularity predicted in the reflection spectrum of silver with or without polymer at *λ* ∼ 376 ± 3 nm (Figure 7a) was due to the tiny feature in the dielectric permittivity of Ag at *λ* ∼ 374 nm (Figure 5b) and practically did not depend on the existence of a polymer. As the thickness of the polymeric films, their absorption, and the incidence angles were varied, the dips in different calculated reflection spectra changed modestly or significantly. Therefore, the latter calculated results were not in a good agreement with the experiment, regardless of the varied materials’ and system’s parameters.

We infer that the excitation of SPPs could be mediated by unintentional subwavelength scatterers, which are common in metallic and polymeric films. Rough features on top of Ag (if significant) can contribute to anomalous reflection dips via field enhancement caused by localized plasmons. The effect of the scattering strength on the excitation of SPPs and the experimentally observed dips in the reflection spectra is the subject of future studies to be published elsewhere.

Alternatively, the observed anomalous dips in the reflection spectra can be attributed to a leaky mode supported by a Berreman-like leaky mode in the light cone [24–27] or other Epsilon Near Zero (ENZ) regime phenomena. In this scenario, realized in the vicinity of the ENZ point of the plasmonic materials, the continuity of the normal component of the electric displacement yields a strong enhancement of the normal component of electric field, that in turn contributes to reduced reflection of the incident light. The exact mechanism for reflection reduction depends on the thickness of the ENZ materials, the permittivity of surrounding media, dispersion of the (leaky) modes supported by the layer stack, as well as roughness of the surfaces that may affect scattering of light into leaky or guided modes.

## 6 Effect of Au substrates

**Figure 8:**
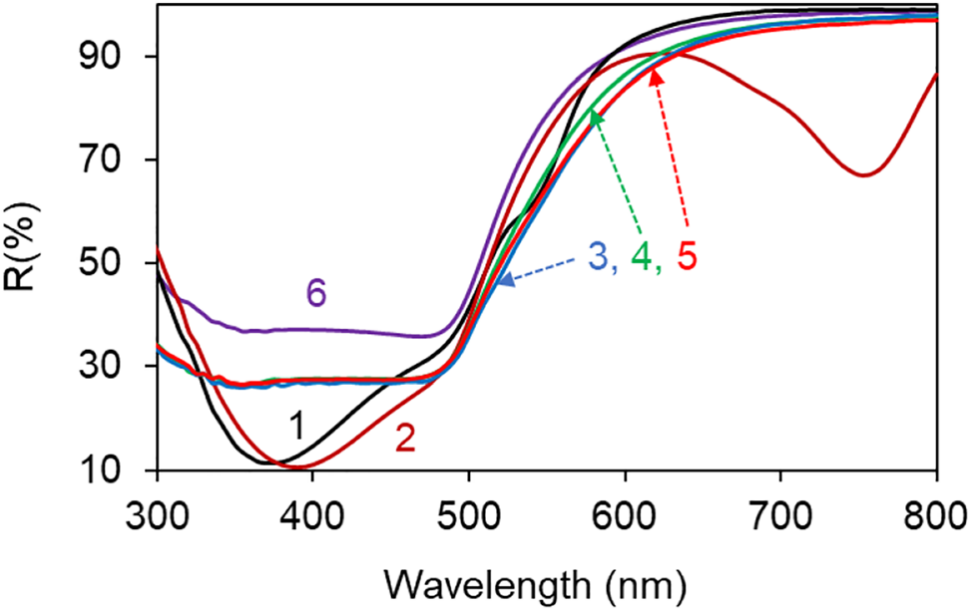
Reflection spectra of Rh590:PMMA (1), HITC:PMMA (2), and undoped polymers PMMA (3), PVP (4), and PS (5) on top of Au, reflection spectrum of Au (6).

**Figure 9:**
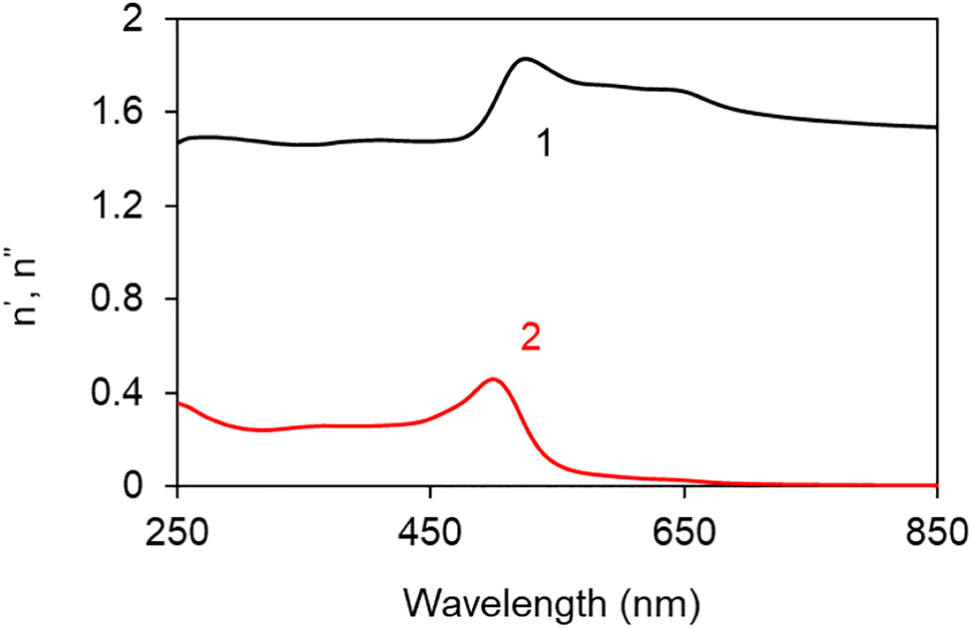
Calculated spectra of real (1) and imaginary (2) parts of refractive indexes of SPPs propagating at the interface between Au and undoped PMMA [20].

The effect of Au substrates on polymers’ reflection spectra was strongly different from that of Ag substrates. Thus, although the dips were observed in reflection spectra of Rh590:PMMA and HITC:PMMA on top of Au, they were larger (deeper), broader, and some of them were strongly shifted to shorter wavelengths, see Figure 8 along with Figure 3a and b. The spectral positions of the reflection dips spread between 290 and 390 nm and the standard deviation was large, *λ* = 325 ± 35 nm in Rh590:PMMA and *λ* = 343 ± 41 nm in HITC:PMMA. Furthermore, no characteristic dips were observed in the reflection spectra of undoped PMMA, PVP, and PS polymers deposited on Au, Figure 8, and no substantial dips were theoretically predicted using the transfer matrix solver, Figure 7b. A modest singularity in the SPP refractive index, predicted theoretically (for dielectric permittivity of Au [20], Figure 5c) at *λ* ∼ 500 nm, Figure 9, cannot explain the dips in the UV reflection observed experimentally. It appears likely that the origin of the reflection dips on top of Au substrate is different from that on top of Ag substrate. This is the subject of a separate study to be published elsewhere.

## 7 Summary

To summarize, we have studied reflection spectra of dye-doped and undoped polymers spin coated onto Ag and Au substrates and found anomalous dips in the UV spectral range. On top of Ag substrates, the *λ* ∼ 375 nm dips were observed in undoped PMMA, PVP, and PS polymers as well as PMMA doped with Rh590 and HITC laser dyes. The dips seemed to be irrelevant to the S_0_–S_1_ and S_0_–S_2_ transitions in Rh590 and HITC. At the same time, the spectral positions of the anomalous reflection dips were close to singularities in the spectra of refractive indexes of SPPs propagating at the interface between Ag and polymer. The latter singularities can tentatively explain the *λ* ∼ 375 reflection dips in the spectra, if the scattering of the Ag and polymeric films is large enough to launch SPP without any prism or grating. Alternatively, the observed anomalous dips in the reflection spectra can be attributed to a Berreman-like leaky mode in the light cone [24–27] or strong field enhancement in the Epsilon Near Zero (ENZ) regime.

The dips observed in reflection of Rh590:PMMA and HITC:PMMA on top of Au, were more pronounced than those on Ag, broader, shifted to shorter wavelengths, and their spectral positions had large standard deviations. Furthermore, no significant anomalous dips were observed in the reflection spectra of undoped PMMA, PVP, and PS polymers. A modest singularity in the SPP refractive index, predicted theoretically at *λ* ∼ 500 nm for Au, cannot explain the dips in the UV reflection spectra observed experimentally. It appears likely that the origin of the reflection dips on top of Au substrate is different from that on top of Ag substrate.
